# Pituitary tumor apoplexy associated with extrapontine myelinolysis during pregnancy: A case report

**DOI:** 10.1097/MD.0000000000025075

**Published:** 2021-03-12

**Authors:** Wenfeng Ye, Wenjie Huang, Linlin Chen, Changfang Yao, Shiying Sheng, Zhengyu Liu, Chunyan Xue, Wei Xing

**Affiliations:** a Department of Obstetrics and Gynecology; b Department of Radiology; c Department of neurology; d Department of endocrinology, The Third Affiliated Hospital, Soochow University, Changzhou, Jiangsu, China.

**Keywords:** extrapontine myelinolysis, hyponatremia, pituitary tumor apoplexy, pregnancy

## Abstract

**Rationale:**

Pituitary tumor apoplexy (PTA) is a rare clinical syndrome which requires urgent diagnosis and treatment due to its life-threatening consequences. Management of undiagnosed pituitary tumor before pregnancy is a problem during pregnancy.

**Patient concerns:**

We reported a case with PTA which was not diagnosed before pregnancy presenting with vomiting associated with hyponatremia during the third trimester. After supplying the sodium the patient presented with dysarthria and hemiplegia.

**Diagnoses:**

MRI examination showed PTA accompanied with extrapontine myelinolysis (EPM).

**Interventions:**

The patient was given hydrocortisone according to the symptoms gradually to taper off dose, at the same times oral levothyroxine therapy (25μg/day) was given.

**Outcomes:**

The patient delivered a healthy baby via cesarean section at hospital at 38 + 1 week of gestation. We performed MRI examination regularly and the tumor regressed significantly 8 months postpartum.

**Lessons:**

We reported a case as PTA associated with EPM. Headache during pregnancy is often nonspecific, so careful medical history inquiry is very important.

## 1 Introduction

Pituitary tumor apoplexy (PTA) is a rare clinical syndrome with an estimated prevalence of 6.2 cases per 100,000 persons, which requires urgent diagnosis and treatment due to its life-threatening consequences.^[1]^ Management of undiagnosed pituitary tumors before pregnancy is a problem during pregnancy and faces some safety issues including potential tumor growth and apoplexy which are very low and will be confronted with a major concern by the clinical doctors.^[2]^ Pregnancy is one of the risk factors for pituitary apoplexy (PA) because the enlarged size of the pituitary gland, increased blood flow in the gland and increased hormones which stimulate the gland and pituitary tumor.^[3,4]^ The PA occurrence rate during pregnancy is rare so that the diagnosis and treatment is sometimes neglected or delayed.

PTA is characterized by some acute clinical syndrome including headache, nausea, vomiting, visual abnormity, and/or decreased consciousness because of the result of a pituitary tumor infarction or hemorrhage.^[5]^ Nausea and vomiting, as one of the most common symptoms of PTA, is very common during pregnancy and it does not get much attention from the patients and clinicians. Vomiting could cause pregnant women to develop severe electrolyte disorders, among which hypokalemia and hyponatremia occur most frequently. Hyponatremia refers to the serum sodium concentration less than 135 mmol/L, which is one of the most common kinds of water and salt imbalance in clinical practice, accounting for most of hospitalized patients. Hyponatremia can increase the complexity of symptoms and lead to misdiagnosis by the clinicians. There are different treatment principles about of hyponatremia of different degree, and inappropriate treatment such as rapid correction of hyponatraemia in patients may cause demyelination disease of nerve permeability.^[6,7]^ Central pontine myelinolysis (CPM) and extrapontine myelinolysis (EMP) belong to osmotic demyelination syndrome (ODS) which can be caused in the treatment of hyponatremia associated with poor prognosis.^[8]^ The patients with ODS present differently, including acute paralysis, dysarthria and dysphagia.

We report a case with PTA who was not diagnosed before pregnancy presenting with vomiting associated with hyponatremia during the third trimester. After supplying the sodium the patient presented with dysarthria and hemiplegia, MRI examination showed PTA accompanied with EPM and the patient was managed conservatively with a successful outcome. We performed MRI examination regularly and the tumor regressed significantly 8 months postpartum.

## 2 Case report

A 24-year-old woman with a history of vomiting for 3 days was admitted at emergency ward during the 32th week on December 11th, 2018. She complained that she had a history of low fever without measuring body temperature, no abdominal pain, no diarrhea and other discomfort and she did not take it seriously at first because she thought it was common flue or upper respiratory tract infection. But 1 day before, she began to vomit severely associated with fatigue.

Urgent blood arterial gas analysis for the patient showed that serum sodium concentration as high as 111.6 mmol/L, chlorine as 91.3 mmol/L, plasmatic osmolality as 226 m Osm 226/kg. Blood electrolyte test showed that serum sodium concentration was as 116.7 mmol/L, chlorine concentration as 87.3 mmol/L and potassium as 4.52 mmol/L in emergency ward. The patient was given 3% NaCl 400 ml by intravenous infusion. The next day, blood electrolyte retest showed that serum sodium concentration was 126.0 mmol/L (Table 1), so 3% NaCl 400 ml was given by intravenous infusion in the morning again. The patient developed aphasia and hemiplegia with no movement of the right limb in the afternoon suddenly, so she was admitted to the obstetric department for further treatment.

**Table 1:** Clinical detailed results of blood biochemistry measurements.

value	Date	Result (mmol/l)	normal range	Annotation
Osmolality	2018-12-11	226 Osm/kg	280–310mOsm/kg	3%Nacl 400 ml was given
	2018-12-13	252 Osm/kg		
	2018-12-13	280 Osm/kg		
Sodium	2018-12-11	116.7g mmol/l	137–147 mmol/l	3%Nacl 400 ml was given
	2018-12-12	126 mmol/l		3%Nacl 400 ml was given
	2018-12-13	132.7 mmol/l		
	2019-01-01	135.8 mmol/l		
	2019-01-15	132.8 mmol/l		
Potassium	2018-12-11	4.52 mmol/l	3.5–5.3 mmol/l	
	2018-12-12	4.67 mmol/l		
	2018-12-13	4.49 mmol/l		
	2019-01-01	4.4 mmol/l		
	2019-01-15	3.6 mmol/l		
Chloride	2018-12-11	87.3 mmol/l	98–110 mmol/l	3%Nacl 400 ml was given
	2018-12-12	101.6 mmol/l		3%Nacl 400 ml was given
	2018-12-13	107.7 mmol/l		
	2019-01-01	105.7 mmol/l		
	2019-01-15	105 mmol/l		
Fibrinogen	2018-12-13	6.76 g/l	2–4 g/l	Heparin was given
	2018-12-15	3.77 g/l		

After admission at the obstetric department, she was afebrile with 36.6°C on examination, with a heart rate of 101bpm and blood pressure of 122/77 mm Hg. Physical examination revealed that the patient's right nasolabial groove became shallow and her tongue extended to the right direction. Her visual field detection was normal. She was retarded with aphasia associated with hemiplegia in right arms and legs. Her right upper limb power was grade 0/5, right lower limb power was grade 3/5 and the left limb power was 4/5. Her pain sensitivity was lower in the right than that in the left. All her deep tendon reflexes were positive. Her plantar response was positive in the right. The rest of her physical examination was unremarkable. Obstetrical examinations showed a gravid uterus at around 32 weeks of gestation with normal fetal heart rate.

**Figure 1:**
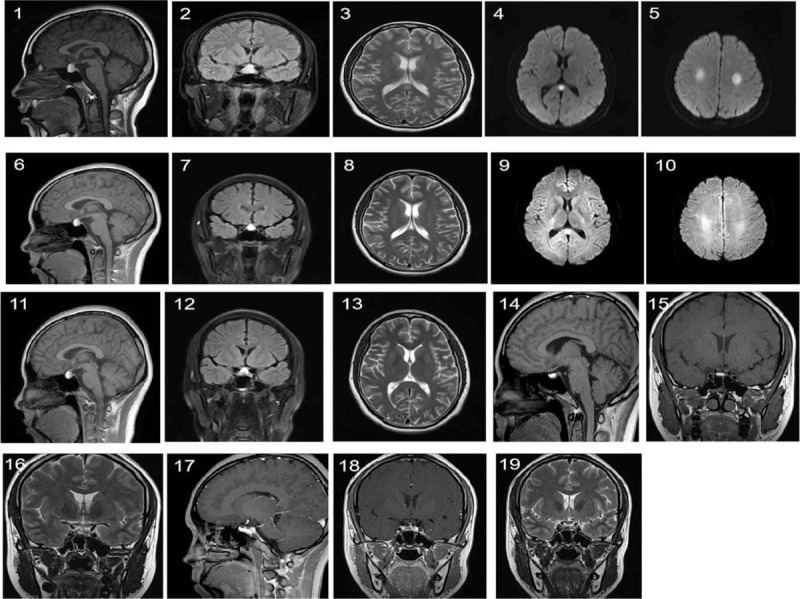
Brain Magnetic resonance imaging of the patient. (A) sagittal T1-weighted images shows a sellar and suprasellar high signal with the size of 15mm × 14mm × 14 mm, in which stratification was demonstrated, (B) coronal FLAIR images shows the high signal appeared as an hourglass, (C) axial T2-weighted images shows hyperintensity in bilateral basal ganglia region and corpus callosum, (D) (E) axial DWI images shows hyperintensity in corpus callosum and bilateral centrum semiovale (picture A–E, during 32 + 1 gestational week on December 12th,2018), (F) (G) shows the size of sellar and suprasellar high signal is reduced to 13mm × 12mm × 13 mm, compared with the previous examination (picture 1, 2),(H) shows the lesion in corpus callosum was slightly smaller, compared with picture 3,(I) (J) the signal of the lesion in corpus callosum and bilateral centrum semiovale was weaker, compared with picture 4, 5 (picture F–J, on December 20th,2018), (K)(L)shows the size of sellar and suprasellar high signal was reduced to 10mm × 11mm × 12 mm, (M)shows the abnormally high signal in bilateral basal ganglia region basically disappeared (picture K–M, on January 18th,2019), (N)(O)(P)shows the size of sellar and suprasellar high signal was further reduced to 8mm × 5mm × 9mm (on April 27th,2019),(Q)(R)contrast enhanced images shows the pituitary gland was almost normal in size, with homogeneous enhancement after enhancement, and pituitary stem was slightly shifted to the left, (S) coronal T2-weighed images shows the pituitary gland appeared as heterogeneous signal (on September 24th,2019).

An emergency cerebral magnetic resonance imaging (MRI) was performed and a sellar abnormal signal with size about 15 × 14 × 14 mm was revealed, which indicated the PTA. Abnormal signals in corpus callosum, bilateral basal ganglia, and centrum semiovale were seen. There were no obvious abnormalities in magnetic resonance angiography and venography (Fig. 1). Immediate laboratory testing revealed as follow: PH 7.31, blood oxygen saturation 98.8%, CO_2_ partial pressure 23.10 mm Hg, plasmatic osmolality 252 Osm 226/kg, blood glucose 2.8 mmol/l, lactic acid <1 mmol/L by blood arterial gas analysis. Blood routine showed hemoglobin of 11.5 mg/dl, PLT of 248 × 10^9^/L. Biochemistry test showed Na of 131.6 mmol/L, CO_2_ of 12.3 mmol/L, BUN of 1.0 mmol/L, creatinine of 45U mol/L, blood amylase of 63μ/L, blood lipase of 119μ/L, fibrinogen of 6.01 g/L (Table 1).

Further hormonal examination results after admission were as follows: plasma prolactin (PRL) level of 104.71 ng/ml (3.34–26.72 ng/ml), TSH level of 1.49 mIU/ml (0.3–5.5 mIU/ml), fT4 level of 11.26 pmol/L (11.46–23.17 pmol/L), fT3 level of 3.25 pmol/L (2.8–7.1 pmol/L), ACTH level (8AM) of 9.53 pg/ml (6–40 pg/ml), ACTH level (4PM) of 7.12 pg/ml (3–30 pg/ml), and cortisol level (8AM):15.46 μg/dl (6.7–22.8), cortisol level (4PM) of >61.4 μg/dl (0–10 μg/dl), growth hormone of 0.65 ng/ml (0.06–5ng/ml) (Table 2). High cortisol level of 4PM was associated with hydrocortisone taking.

**Table 2:** Clinical detailed results of hormonal measurements.

value	Date	Result (mmol/l)	normal range	Annotation
TSH	2018-12-13	1.49 UIU/ml	0.3–5.5 UIU/ml	2019-01-23 delivery
	2018-12-18	3.03 UIU/ml		
	2019-01-08	3.67 UIU/ml		
	2019-01-25	6.97 UIU/ml		
	2019-02-13	1.39 UIU/ml		
	2019-09-18	4.06 UIU/ml		
	2019-11-17	2.88 UIU/ml		
FT4	2018-12-13	11.26 pmol/l	11.46–23.17pmol/l	
	2018-12-18	11.68 pmol/l		
	2019-01-08	12.5pmol/l		
	2019-01-25	13.09 pmol/l		
	2019-02-13	14.99 pmol/l		
	2019-09-18	11.7 pmol/l	12–22 pmol/l	
	2019-11-17	11.57 pmol/l		
FT3	2018-12-13	3.25 pmol/l	2.8–7.1pmol/l	
	2018-12-18	3.11 pmol/l		
	2019-01-08	3.27 pmol/l		
	2019-01-25	3.03 pmol/l		
	2019-02-13	4.6 pmol/l		
	2019-09-18	4.46 pmol/l	3.1–6.8pmol/l	
	2019-11-17	4.22 pmol/l		
Cortisol	2018-12-14	8AM:15.46μg/dl	6.7–22.8 μg/dl	Cortisol 4PM (2018-12-14) after hydrocortisone using
	2018-12-14	4PM:>61.4μg/dl	0–10 μg/dl	
	2019-01-25	8AM:8.87μg/dl	6.7–22.8 μg/dl	
	2019-01-25	4PM:5.58μg/dl	0-10 μg/dl	
	2019-09-18	8AM:7.37μg/dl	6.7–22.8 μg/dl	
	2019-09-18	4PM:2.2μg/dl	0–10 μg/dl	
ACTH	2018-12-14	8AM:9.53 pg/ml	6–40 pg/ml	
	2018-12-14	4PM:7.12 pg/ml	3–30 pg/ml	
	2019-01-26	8AM:16.6 pg/ml	6–40 pg/ml	
	2019-01-26	4PM:7.36 pg/ml	3–30 pg/ml	
	2019-09-18	8AM:19.95pg/ml	6–40 pg/ml	
	2019-09-18	4PM:11.66pg/ml	3–30 pg/ml	
Prolactin	2018-12-13	104.71 ng/ml	3.34–26.72 ng/ml	Postpartum no breastfeeding
	2018-12-18	108.40 ng/ml		
	2019-01-25	110.55 ng/ml		
	2019-02-13	59.06 ng/ml		
	2019-07-19	50.76 ng/ml		
	2019-09-18	48.80 ng/ml		
	2020-01-17	44.59 ng/ml		
Growth hormone	2018-12-13	0.65 ng/ml	0.06–5 ng/ml	
	2019-02-13	0.06 ng/ml		
	2019-09-18	0.87 ng/ml		
Insulin-like growth factor	2019-02-13	195.5 ng/ml	60–350 ng/ml	
	2019-09-18	176 ng/ml		

A multidisciplinary team was organized including neurology, neurosurgery, endocrinology, ICU and obstetric department. PA with pituitary insufficiency were considered which caused hyponatremia and sodium supplied rapidly for EPM induction with hemiplegia and aphasia. The patient was given hydrocortisone according to the symptoms gradually to taper off dose, at the same times oral levothyroxine therapy (25μg/day) was given. The symptoms improved after 2 days and the patient could speak and recovered the movement of limbs. Her medical history was got which included that she had been irregular menstruation from the menarche at 14-year-old. In 2 years before pregnancy (August 23, 2016) she had taken hormone test and results were nearly normal. After that she had not gone to the hospital until she was pregnancy. She complained severe headache for the entire pregnancy and got the drug for painkiller at the early pregnancy. After taking medications, her symptoms did not improve and because of worrying about the outcomes of drugs on fetal, so she stopped the drug. After using hydrocortisone and levothyroxine, the symptoms including headache and vomiting disappear, MRI after 8 days at admission showed that abnormal signal in the splenium of the corpus callosum, bilateral basal ganglia, and centrum semiovale were weaker significantly. She delivered a healthy baby via cesarean section at hospital at 38 + 1 week of gestation (January 23, 2019). The baby birth weight was 2850 grams. The Apgar score was 9’ and 9’at first and fifth minutes, respectively. The blood volume during operation was 1000 ml. The patient did not have breastfeeding after delivery.

Postpartum, the patient did not have headache and vomiting except her irregular menstruation. The pituitary tumor was significantly regressive by MRI (Fig. 1). Meanwhile the blood hormone examination showed that PRL was decreased gradually of 44.59ng/ml (January 17, 2020). Thyroid function tests, serum cortisol, ACTH, growth hormone, and IGF-1 level showed normally. This study was approved by the Ethics Committee of Soochow University. The patient agreed to authorize us to share the figures and the experiences during the treatment procedure in our department. Informed consent was obtained.

## 3 Discuss

Gestational pituitary tumor apoplexy is a rare disease. Considering the situation of lack of consensus, it is difficult to manage the PA cases. So, every patient should be assessed and managed carefully. Especially for some cases with pituitary tumor which was not found before pregnancy it is difficult for timely treatment.

## 4 Clinical signs and diagnosis

PA occurs in patients which are asymptomatic previously in 60% to 80% of cases.^[9]^ The main symptoms of PTA include headache, nausea, visual impairment among which headache occurs in 95% of cases and vomiting in 69% of that.^[10,11]^ Due to the atypical symptoms, misdiagnosis is often caused and serious clinical complications are resulted in.^[12]^

Before pregnancy most of the patients with pituitary tumor have the history of irregular menstruation because of the elevated sexual hormones such as prolactin. Headache is one of the most common symptoms of PTA during pregnancy. Our patient complained of headache, but there was no effect after taking painkiller drugs. This symptom provided us with certain signals, but the clinician did not pay enough attention to it, because headache is common during pregnancy, associated with emotion such as stress, lack of sleep, depression, and malnutrition. In addition pregnant women themselves often fear that drugs for painkiller might affect the fetus, so simple headaches are not taken seriously.^[13]^ Vomiting is one of the symptoms of PTA during pregnancy. The case complained of vomiting for 3 days, but emergency department only routinely conducted blood electrolyte examination, and immediately gave a large amount of sodium supplement treatment after the discovery of hyponatremia. Only the patient had symptoms with aphasia and hemiplegia on the next day, the attention was aroused by the clinicians. Similarly there are many reasons for simple vomiting during pregnancy and clinicians often pay more attention to electrolyte disorders than the reason of hyperemesis. The symptoms including headache and vomiting are caused by the increased intracranial pressure due to PTA and meningeal irritation.^[9,10]^

MRI examination is necessary for the diagnosis of PA and EPM. The precise mechanism underlying EPM and CPM remains elusive. It involves a patient with hyponatremia, in whom compensatory cellular expansion offsets the reduced plasma osmotic pressure. Thereafter, any rapid change in osmosis in the opposite direction, usually caused by hypertonic fluid, causes the swollen cells to shrink, leading to osmotic demyelination. The mechanism of cellular expansion relies upon the generation of osmoses such as taurine, glycine, glutamine, sorbitol, and inositol.^[14]^ Our patient was given the rapid sodium treatment to correct hyponatremia which resulted in transient hemiplegia and aphasia. MRI examination may reveal abnormal signal, which could be considered the acute cerebral infarction if not combined with clinical history or lack of understanding of EMP and may lead to misdiagnosis of clinical doctors.

Traditional CPM was thought as pathological features about pons base symmetrical demyelination of nerve fibers, later it was found that outside parts of the pons also could appear the same change, known as the EPM. Generally, both of them have a history of rapid correction of hyponatremia. EPM without CPM was rare, which could result in misdiagnosis. The hallmark of EPM has the striking T2 signal abnormalities with lesions most commonly occurrence in the cerebellum, the lateral geniculate body, the external and extreme capsule, basal ganglia, thalamus, gray-white junction of the cerebral cortex, and the hippocampi.^[15]^ Although rare, lesions have been described in the spinal cord, amygdala, anterior commissure, optic tracts, and the subthalamic nuclei.^[6,8]^

## 5 Treatment

Management of PA has 2 choices: surgery and conservative medication. When pregnancy is confirmed, most of the women with pre-existing pituitary tumor choose to stop dopamine agonist generally,^[16]^ although the knowledge about pregnancy outcomes for women who have become pregnant while taking bromocriptine or cabergoline is widening. Large adenoma size, pregnancy, and cessation of cabergoline could lead to PA. There are no clear guidelines for the management of PA during pregnancy. In the case of prolactioma and non-stroke symptomatic tumor growth, most of them are recommended reactivating dopamine agonist as first-line therapy, as this is generally considered to be less risky to the mother and fetus than surgical intervention. Half of the patients were treated conservatively with pituitary hormone replacement therapy when necessary, few cases were treated with dopamine agonists.^[17,18]^

There are no randomized controlled trials comparing the effects of surgical and conservative treatment in pregnant women with PA. However, surgery seems appropriate for patients who fail to respond to conservative treatment or cannot tolerate dopamine agonists.^[16]^ Neurosurgical intervention should be considered in cases with persistent visual field defects or deteriorating level of consciousness. If operation, it is emphasized that surgical excision should be performed by experienced neurosurgeons, not by on-call surgeons.

The main conservative treatment with PTA during pregnancy is to provide fluid and electrolyte balance and high dose glucocorticoid for emergency condition. Glucocorticoid is the most commonly used hormonal drug. In addition, if combined with thyroid insufficiency and adrenal insufficiency, it can be supplemented according to the examination results and clinical symptoms. About the CPM and EPM caused by fast natrium replenishment, sodium supplementation must be controlled. After the treatment, the symptoms of aphasia and hemiplegia of this case were rapidly improved and the effect of conservative treatment was obvious. Hypopituitarism is an important complication of apoplexy including hypothyroidism, hypoadrenalism and hyperprolactinaemia and may be missed if not carefully investigated.^[5]^ Therefore, clinicians must pay attention to the occurrence of hypophysis dysfunction and give related hormone therapy in time.

## 6 Prognosis

The risk of pituitary tumor development will increase during pregnancy. The prognosis of PA during pregnancy depends on the timely diagnosis and clinical management. In general, early symptoms such as headache and vomiting should be pay attention. Careful medical history inquiry by the clinician, timely physical examination, MRI application and hormone replacement therapy as soon as possible can significantly improve the therapeutic effect.

## 7 Conclusion

In general, in the case of pregnancy, the diagnosis of PTA can be challenging and confused with other complex situations, such as preeclampsia. MRI is one of the most sensitive examination, revealing to confirm diagnosis of PTA and/or necrosis of part of adrenocorticotropic hormone deficiency and adrenal insufficiency. If not treated, it is a potentially life-threatening disease for both mother and fetus. Headache during pregnancy is often nonspecific, so it is easy to be omitted by clinicians. Therefore, careful medical history inquiry is very important. The patients need to be re-evaluated if chronic headaches do not ease. A multidisciplinary team consisting of neurology, neurosurgery, endocrinology, ICU, and obstetric department is important in deciding the optimal treatment. At the same time, maternal desires should be taking into consideration.

## Author contributions

**Conceptualization:** Wenfeng Ye, Linlin Chen.

**Data curation:** Shiying Sheng, Zhengyu Liu.

**Investigation:** Wenfeng Ye, Linlin Chen, Changfang Yao, Chunyan Xue.

**Project administration:** Wenfeng Ye, Chunyan Xue, Wei Xing.

**Supervision:** Wei Xing.

**Validation:** Changfang Yao, Shiying Sheng, Zhengyu Liu, Chunyan Xue, Wei Xing.

**Visualization:** Wenjie Huang, Linlin Chen, Changfang Yao, Zhengyu Liu, Wei Xing.

**Writing – original draft:** Wenfeng Ye, Wenjie Huang.

**Writing – review & editing:** Wei Xing.

## References

[1] Fernandez A Karavitaki N Wass JAH. Prevalence of pituitary adenomas: a community-based, cross-sectional study in Banbury (Oxfordshire, UK). Clin Endocrinol 2010;72:377–82. 10.1111/j.1365-2265.2009.03667.x 19650784

[2] Molitch ME. Prolactinoma in pregnancy [J]. Best practice & research. Clin Endocrinol Metabol 2011;25:885–96. 10.1016/j.beem.2011.05.011 22115164

[3] Bi WL Dunn IF Laws ER Jr. Pituitary apoplexy. Endocrine 2015;48:69–75. 25063308 10.1007/s12020-014-0359-y

[4] Giritharan S Gnanalingham K Kearney T. Pituitary apoplexy - bespoke patient management allows good clinical outcome. Clin Endocrinol 2016;85:415–22. 10.1111/cen.13075 27038242

[5] Randeva HS Schoebel J Byrne J. Classical pituitary apoplexy: clinical features, management and outcome. Clin Endocrinol 1999;51:181–8. 10.1046/j.1365-2265.1999.00754.x 10468988

[6] Singh TD Fugate JE Rabinstein AA. Central pontine and extrapontine myelinolysis: a systematic review. Eur J Neurol 2014;21:1443–50. 25220878 10.1111/ene.12571

[7] Abbott R Silber E Felber J. Osmotic demyelination syndrome. BMJ Clin Res Ed 2005;331:829–30. 10.1136/bmj.331.7520.829 PMC1246086 16210283

[8] Alleman AM. Osmotic demyelination syndrome: central pontine myelinolysis and extrapontine myelinolysis. Sem Ultrasound CT MR 2014;35:153–9. 10.1053/j.sult.2013.09.009 24745890

[9] Nawar RN AbdelMannan D Selman WR. Pituitary tumor apoplexy: a review. J Intens Care Med 2008;23:75–90. 10.1177/0885066607312992 18372348

[10] Murad-Kejbou S Eggenberger E. Pituitary apoplexy: evaluation, management, and prognosis. Curr Opin Ophthalmol 2009;20:456–61. 19809320 10.1097/ICU.0b013e3283319061

[11] Bills DC Meyer FB Laws ER Jr. A retrospective analysis of pituitary apoplexy. Neurosurgery 1993;33:602–9. 8232799 10.1227/00006123-199310000-00007

[12] Lambert K Rees K Seed PT. Macroprolactinomas and nonfunctioning pituitary adenomas and pregnancy outcomes. Obstetrics gynecol 2017;129:185–94. 10.1097/AOG.0000000000001747 27926659

[13] Negro A Delaruelle Z Ivanova TA. Headache and pregnancy: a systematic review. J Headache Pain 2017;18:106–106. 29052046 10.1186/s10194-017-0816-0 PMC5648730

[14] Lien YH Shapiro JI Chan L. Study of brain electrolytes and organic osmolytes during correction of chronic hyponatremia. Implications for the pathogenesis of central pontine myelinolysis. J Clin Invest 1991;88:303–9. 2056123 10.1172/JCI115292 PMC296033

[15] Juergenson I Zappini F Fiaschi A. Teaching neuroimages: neuroradiologic findings in pontine and extrapontine myelinolysis: clue for the pathogenesis. Neurology 2012;78:e1–2. 22201117 10.1212/WNL.0b013e31823ed0b5

[16] Melmed S Casanueva FF Hoffman AR. Diagnosis and treatment of hyperprolactinemia: an endocrine society clinical practice guideline. J Clin Endocrinol Metabol 2011;96:273–88. 10.1210/jc.2010-1692 21296991

[17] Motivala S Gologorsky Y Kostandinov J. Pituitary disorders during pregnancy. Endocrinol Metabol Clin N Am 2011;40:827–36. 10.1016/j.ecl.2011.08.007 22108282

[18] Molitch ME. Prolactinomas and pregnancy. Clin Endocrinol 2010;73:147–8. 10.1111/j.1365-2265.2010.03823.x 20550542

